# Identification and Pathogenicity Analysis of a Novel Fibrinogen Bβ Chain p.Gly293Val Variant Causing Hypofibrinogenemia

**DOI:** 10.14740/jh2183

**Published:** 2026-04-06

**Authors:** Xiao Li Cheng, Lin Zhu, Yi Juan Xin, Liu Yang, Jia Yun Liu

**Affiliations:** a Department of Clinical Laboratory Medicine, Xijing Hospital, Fourth Military Medical University, Xi’an, Shaanxi, China

**Keywords:** Hypofibrinogenemia, Fibrinogen, *FGB*, Variant

## Abstract

**Background:**

Hypofibrinogenemia is a rare bleeding disorder characterized by excessive bleeding, impaired wound healing, and elevated perioperative risk. It most commonly results from pathogenic variants in the *FGB* gene. This study aimed to analyze the clinical phenotypes and genetic variants in a family with hypofibrinogenemia and explore its molecular pathogenic mechanisms.

**Methods:**

Fibrinogen (Fg) activity (Fg:C) was measured using the Clauss method and the prothrombin time (PT)-derived method, and Fg antigen (Fg:Ag) levels were determined by enzyme-linked immunosorbent assay (ELISA). Fg polymerization capacity was evaluated via a thrombin-induced Fg polymerization assay, and Fg levels and function were assessed using thromboelastography. Sanger sequencing was performed to screen for variants in all exons and flanking regions of the *FGA*, *FGB*, and *FGG* genes. Multiple *in silico* tools, including ClustalX-2.1-win, MutationTaster, PolyPhen-2, PROVEAN, I-Mutant 2.0 and Swiss-Pdb Viewer, were used to assess the conservation of the variation sites and their impact on protein structure and function. The pathogenicity of the variation sites was evaluated according to the American College of Medical Genetics and Genomics (ACMG) standards and guidelines for the interpretation of sequence variants.

**Results:**

The proband and affected members exhibited prolonged thrombin time (TT), reduced Fg:C and Fg:Ag levels, and a hypocoagulable thromboelastography profile. Notably, Fg polymerization kinetics remained preserved, consistent with hypofibrinogenemia rather than dysfibrinogenemia. Genetic analysis identified a heterozygous missense variant *FGB* c.878G>T (p.Gly293Val) segregating with the phenotype. This variant was absent from population databases, located at a highly conserved residue, and predicted to be deleterious by multiple *in silico* tools. Protein structural modeling indicated local conformational disturbance. The variant was classified as likely pathogenic following the 2015 ACMG/Association for Molecular Pathology (AMP) standard guidelines.

**Conclusions:**

The *FGB* p.Gly293Val variant may cause a significant decrease in Fg:C and Fg:Ag by disrupting the structure and function of the Fg protein.

## Introduction

Hereditary fibrinogen (Fg) disorders are rare bleeding disorders caused by pathogenic variants in *FGA*, *FGB*, and *FGG* [1]. Clinical severity varies widely and is strongly associated with zygosity [2]. Homozygous or compound heterozygous individuals typically present with severe, life-threatening bleeding from early infancy, including umbilical stump bleeding, intracranial hemorrhage, or major post-traumatic bleeding. In contrast, heterozygous carriers usually show mild manifestations, such as easy bruising, menorrhagia, or mucosal bleeding, and may even be asymptomatic until hemostatic challenge. These disorders are classified into two main types: type I (hypofibrinogenemia and afibrinogenemia), characterized by concordant reduction in Fg antigen (Fg:Ag) and functional activity (Fg:C), and type II (dysfibrinogenemia and hypodysfibrinogenemia), characterized by disproportionately low Fg:C despite normal or near-normal Fg:Ag levels [3]. The structure–function relationship within the Fg molecule is critical: variants that impair synthesis or secretion lead to type I deficiency, whereas those that affect functional domains cause type II dysfunction. This direct link between genetic variation, protein function, and clinical phenotype provides the foundation for understanding disease mechanisms.

Fg is a 340 kDa complex glycoprotein synthesized primarily in hepatocytes, acting as the final substrate in the coagulation cascade [4]. Its functional hexameric structure (Aα Bβ γ)_2_ is encoded by the *FGA*, *FGB*, and *FGG* genes clustered on chromosome 4q31.3 [5]. Critical functional domains within Fg mediate key coagulation events: thrombin cleavage of fibrinopeptides in the E-domain exposes polymerization sites, while D-domain interactions drive fibrin monomer assembly, and factor XIIIa (FXIIIa)-catalyzed cross-linking stabilizes the clot [6, 7]. In hereditary disorders, pathogenic variants either disrupt Fg synthesis, processing, or secretion (causing type I deficiency) or alter functional domains (e.g., thrombin cleavage, polymerization, or cross-linking sites) leading to type II dysfibrinogenemia [8]. This structure–function relationship directly links the location and nature of genetic defects to functional impairment and clinical phenotype, supporting the classification and heterogeneous manifestations of Fg disorders.

Despite rapid expansion of the Fg variant catalogue via genetic screening, the functional and clinical relevance of many variants remains undefined. Notably, variants affecting the *FGB-*encoded Bβ chain remain relatively understudied compared with those in *FGA* and *FGG*, and limited functional data are available for residues in the β-chain core domain. The glycine (Gly) 293 residue is located in a structurally conserved loop region critical for Fg stability and secretion, and variants at nearby positions such as arginine (Arg) 294 have been linked to hypofibrinogenemia [9, 10]. Therefore, functional characterization of this novel variant is of great value to clarify genotype–phenotype relationships. Here, we report a novel heterozygous *FGB* variant c.878G>T (p.Gly293Val) identified in a Chinese pedigree with hypofibrinogenemia. This variant has not been previously reported or functionally characterized. To clarify its pathogenicity, we performed comprehensive phenotypic analyses using routine coagulation assays, thromboelastography (TEG) and Fg polymerization testing. We further integrated conservation analysis, bioinformatics prediction, and structural modeling to elucidate how this variant alters Fg structure, and function.

## Materials and Methods

### Clinical information collection

**Figure 1:**
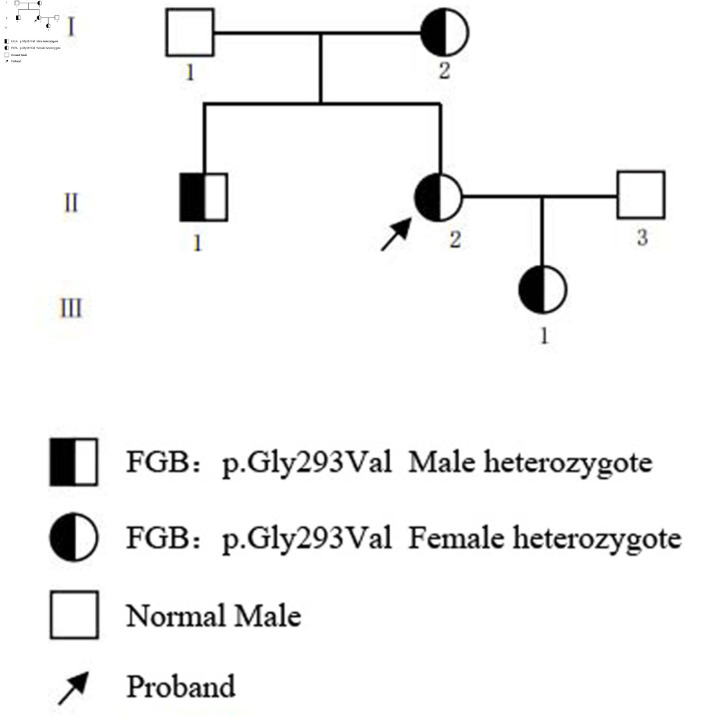
Pedigree of the family with hereditary fibrinogen deficiency.

The proband was a 35-year-old woman who presented to our hospital with a primary complaint of menorrhagia, characterized by prolonged menstrual bleeding (> 7 days), increased menstrual flow, and occasional passage of blood clots, corresponding to an International Society on Thrombosis and Hemostasis-Bleeding Assessment Tool (ISTH-BAT) score of 1. No spontaneous gingival bleeding, epistaxis, ecchymosis, hemarthrosis, or postoperative bleeding was reported. Comprehensive clinical evaluation was performed to rule out secondary causes of bleeding: in addition to excluding hepatic or renal dysfunction, we also eliminated autoimmune diseases, infections, medication use (e.g., anticoagulants, non-steroidal anti-inflammatory drugs), and deficiencies of other coagulation factors (e.g., factors VIII, IX, XI, and von Willebrand factor) as contributing factors. In total, we collected a three-generation pedigree comprising six family members (Fig. 1). The proband’s mother had a long-standing history of menorrhagia before menopause, with similar manifestations of increased menstrual blood loss and prolonged duration, consistent with an ISTH-BAT score of 1. No bleeding or thrombotic symptoms were reported in the other family members (ISTH-BAT score 0). This study was conducted in accordance with the Declaration of Helsinki and approved by the Institutional Review Board of Xijing Hospital, Fourth Military Medical University (Approval No.: KY20252367). Written informed consent was obtained from all participants prior to enrollment.

### Blood sample collection and processing

Peripheral venous blood samples from the proband and participating family members were collected strictly in accordance with standard operating procedures (SOPs) into two separate vacuum tubes containing 0.109 mol/L trisodium citrate anticoagulant (blood-to-anticoagulant ratio of 9:1). This standardized collection approach was implemented to minimize pre-analytical variability, as plasma Fg is an inflammatory response protein sensitive to handling conditions. One tube was used for whole blood TEG analysis. The other tube was centrifuged at 3,000 × g for 15 min at room temperature to obtain platelet-poor plasma (PPP) for subsequent coagulation assays and Fg polymerization studies. The remaining peripheral blood cells were stored at –20°C for genomic DNA extraction.

### Coagulation assays

Routine coagulation parameters were measured using a T711 fully automated coagulation analyzer (Roche Diagnostics, Basel, Switzerland) with manufacturer-matched reagents. Standard clotting assays were employed to determine activated partial thromboplastin time (APTT), prothrombin time (PT) and thrombin time (TT). D-dimer (D-D) and Fg/Fb degradation products (FDPs) were quantified by immunoturbidimetric assays. Fg:C was assessed using both the Clauss method (Fg:C-Clauss) and the PT-derived method (Fg:C-PT-der). Fg:Ag level was measured by an enzyme-linked immunosorbent assay (ELISA) kit (Wuhan Sanying Biotechnology Co., Ltd., Wuhan, China).

### Thrombin-induced Fg polymerization assay

Fg polymerization kinetics were assessed by a turbidimetric assay using a microplate reader (Molecular Devices LLC, San Jose, CA, USA). Citrated PPP samples from the proband and 10 healthy control individuals were used. To eliminate the confounding effect of reduced Fg quantity in hypofibrinogenemia, the Fg concentration in each plasma sample was adjusted and standardized to 0.5 g/L by dilution with Owen’s Kinetic (OK) buffer (50 mM Tris-HCl, 100 mM NaCl, 1 mM CaCl_2_, pH 7.4) before analysis. Fg polymerization was monitored at 37 °C by adding thrombin (1.0 NIH U/mL final concentration). Turbidity was measured at 350 nm every 60 s for 60 min. The resulting turbidity curves were analyzed to determine lag time (LT, time to initial increase), maximum slope (Vmax, maximum polymerization rate), and maximum absorbance (Amax, final clot density). Results from the proband were compared with the mean and range of the control group (10 age- and sex-matched healthy individuals).

### TEG

Global hemostatic function was assessed by TEG using a CFMS TEG Analyzer (Lepu Medical Technology Co., Ltd., Beijing, China) according to the manufacturer’s instructions. Briefly, 340 µL of citrated whole blood was recalcified with 20 µL of 0.2 M CaCl_2_ in a disposable cup to initiate clot formation. Key TEG parameters were continuously recorded, including reaction time (R), kinetics time (K), alpha angle (α), maximum amplitude (MA), coagulation index (CI), percentage of clot lysis at 30 min (LY30), and estimated percent lysis (EPL).

### Genetic sequencing

Genomic DNA was extracted using an NP968 automated nucleic acid extraction instrument (Tianlong Science and Technology Co., Ltd., Xi'an, Shaanxi, China) with its matching reagents. All coding exons, flanking intronic regions, and the 5’- and 3’-untranslated regions (UTRs) of the *FGA* (NM_021871.4), *FGB* (NM_005141.5), and *FGG* (NM_000509.6) genes were amplified by conventional polymerase chain reaction (PCR) on a 9700 Thermal Cycler (Applied Biosystems, Foster City, CA, USA). The primer sequences and thermal cycling conditions are shown in Table 1. PCR products were subjected to Sanger sequencing on an Applied Biosystems 3730xl DNA Analyzer (Thermo Fisher Scientific Inc., Waltham, MA, USA). Sequence chromatograms were analyzed using Chromas software (Technelysium Pty Ltd, South Brisbane, QLD, Australia). Detected variant was confirmed by reverse-strand sequencing. Subsequently, the corresponding exons were specifically amplified and sequenced in all available family members.

**Table 1:** PCR Primers for the *FGA*, *FGB* and *FGG* Gene

Target region	Primer sequence (5'→3')	Annealing temperature (°C)
Exon A1	5'-CTTATAGAAAGCCTTCAGGG-3'	56 °C
	5'-GTGGAATAAACACCAGAGAG-3'	
Exon A2	5'-ATCTCTGTGAGAGTGCCATC-3'	56 °C
	5'-TTTCTGGGACCAATCAGGTC-3'	
Exon A3	5'-TGAATCTGAGAGATAGATCCTTACTG-3'	56 °C
	5'-TTTATTTAGGATTTTTGTTGTTTCTG-3'	
Exon A4	5'-CAGCAGCTACTTCAATAACC-3'	56 °C
	5'-GTGCATAACTATCGCCTTCC-3'	
Exon A5-1	5'-ACCAGGAACTCAATAGACGTAG-3'	56 °C
	5'-AGTTCCAGCTTCCAGCACTG-3'	
Exon A5-2	5'-TATGGAACCGGATCAGAGAC-3'	56 °C
	5'-TGACACCTCTTCAAATGTGCC-3'	
Exon A5-3	5'-ATCTGGAAGTTTTAGGCCAG-3'	56 °C
	5'-TGACAAACTCTCCTAACATAGG-3'	
Exon A5-4	5'-TGATGAAGCTGCCTTCTTCG-3'	56 °C
	5'-AATGACGTGTAACAGAGAG-3'	
Exon A6-1	5'-AGCCGTGCCTATCTTTGTAAAG-3'	56 °C
	5'-TTCATAGGAGGAGACTTGGAG-3'	
Exon A6-2	5'-TCTGGCTAGGCAATGACTAC-3'	56 °C
	5'-AAGACAGAGTGCTCCCATTC-3'	
Exon B1	5'-CTGAAGTCATTCCTAGCAGAG-3'	56 °C
	5'-CAATAAGTCAGAGGTTAACAATT-3'	
Exon B2	5'-ATGAGGGTGTTGGAATAGTTAC-3'	56 °C
	5'-CAATAAGTCAGAGGTTAACAATT-3'	
Exon B3	5'-AATGTCCATGACCCAAATC-3'	56 °C
	5'-ATGCTATTCACTAACCCAGAA-3'	
Exon B4	5'-ATTCTCAGAAAATCAAAATTGTAT-3'	56 °C
	5'-ATGATCTGTTGGGAAAATCC-3'	
Exon B5	5'-AAACTGGGTTGGACTTAC-3'	56 °C
	5'-ATTACAGGTATGAGCCAC-3'	
Exon B6	5'-ATGGACAGGGGATTCAGATA-3'	56 °C
	5'-TAAAACAGGCTTCCAACAAT-3'	
Exon B7	5'-GGCAGTTTTTAGTTTCCCA-3'	56 °C
	5'-ACAGTAAGTGCCCAGGAAGT-3'	
Exon B8	5'-CTTGACCACCGTAGTTCTGT-3'	56 °C
	5'-GCTTGAGAGTTTTAGAGGAATA-3'	
Exon G1+2	5'-GTGCAAAATCTGGGAACC-3'	55 °C
	5'-AAAGTTACAAGTGCCAGATGA-3'	
Exon G3+4	5'-TAAATATCATCTGGCACTTGTA-3'	55 °C
	5'-ACTTCTATCTCTACTATGCTCAAC-3'	
Exon G5	5'-TATTTGTGTTGGGAGTTGAGAC-3'	55 °C
	5'-TTACTTTTCACATCAGCATTCC-3'	
Exon G6	5'-AAGGTTATATTGGGATTAGGTT-3'	55 °C
	5'-TTGCTTATTAGTTATGTGGTCTT-3'	
Exon G7	5'-GCAACCCTAAGAAGTAACCAT-3'	55 °C
	5'-CCAAGAACCAAACAGACTCC-3'	
Exon G8	5'-CCTACGAAAGAGGGAACT-3'	55 °C
	5'-ATGCCAAACTACTGGATA-3'	
Exon G9	5'-ATGCACTTCGTAATAGACAGC-3'	55 °C
	5'-CTTTGTGGGTCAATAGAAGTTA-3'	
Exon G10	5'-TGTCATTTATTTTGTCTTCGTA-3'	55 °C
	5'-ATGGGGTCTTTTGCTCTTA-3'	

### Amino acid conservation analysis

Evolutionary conservation of the mutated amino acid residues was analyzed by multiple sequence alignment. Using the Clustal X software (version 2.1), the amino acid sequences encompassing the variant site were aligned with their orthologs from 10 vertebrate species, including *Mus musculus* (house mouse), *Rattus norvegicus* (brown rat), *Pan troglodytes* (chimpanzee), *Macaca mulatta* (rhesus monkey), *Bos taurus* (cattle), *Canis lupus familiaris* (dog), *Gallus gallus* (chicken), *Xenopus tropicalis* (tropical clawed frog), and *Danio rerio* (zebrafish). Corresponding amino acid sequences were retrieved from the HomoloGene database. The degree of conservation across species was assessed visually from the alignment output.

### Bioinformatic analysis of variant

To predict the potential functional impact of the identified amino acid variant, we employed a suite of *in silico* prediction tools. Four distinct algorithms were utilized: MutationTaster, PolyPhen-2, PROVEAN, and I-Mutant 2.0. All analyses were performed using the canonical *FGB* transcript (NM_005141.5) and its corresponding protein sequence (P02675) as the reference.

### Structural modeling of amino acid variant

The potential structural consequences of the amino acid substitution were analyzed using Swiss-PdbViewer (version 3.7). The wild-type three-dimensional structure of the Fg Bβ chain was obtained from the Protein Data Bank (PDB ID: 3GHG). The amino acid substitution was introduced *in silico*, and the resulting mutant model was energy-minimized using the built-in GROMOS 43B1 force field within the software. The local structural environment, including changes in atomic contacts, potential hydrogen bonds, and steric clashes, was compared between the wild-type and mutant models to infer the impact of the variant on protein stability or interactions.

### Pathogenicity assessment

Variant pathogenicity was classified in accordance with the guidelines established by the American College of Medical Genetics and Genomics (ACMG).

## Results

### Coagulation screening

The proband exhibited a characteristic coagulation profile consistent with hypofibrinogenemia, featuring prolongation of APTT (52.9 s), PT (14.4 s), and a markedly prolonged TT (32.0 s). Fg:C was significantly reduced, measuring 0.76 g/L by the Clauss assay and 0.96 g/L by the PT-derived method, resulting in an Fg:C-Clauss/Fg:C-PTder ratio of 0.79. The Fg:Ag was correspondingly low at 0.99 g/L. Family screening revealed that her mother, brother, and daughter shared an identical phenotype, with prolonged APTT, PT, and TT, alongside concordantly reduced levels of Fg:C-Clauss, Fg:C-PT-der, and Fg:Ag, all approximately 1.0 g/L. In contrast, the proband’s father and husband displayed normal values for APTT, PT, TT, Fg:C, and Fg:Ag (Table 2).

**Table 2:** Phenotypes and Genotypes of the Family With Hereditary Fibrinogen Deficiency

	Age	APTT (s)	PT (s)	TT (s)	D-D (mg/L FEU)	FDPs (µg/mL)	Fg:C (Fg-Clauss assay) (g/L)	Fg:C (Fg-PT der assay) (g/L)	Fg-Clauss/Fg-PT der	Fg:Ag (g/L)	FGB: p.Gly293Val
II2(proband)	35	52.9↑	14.4↑	32.0↑	0.27	4.03	0.76↓	0.96↓	0.79	0.99↓	+
I1 (father)	63	32.5	8.9	16.7	0.4	4.5	2.98	3.1	0.96	3.06	–
I2 (mother)	62	59.9↑	15.1↑	33.2↑	0.32	3.98	0.89↓	1.01↓	0.88	1.03↓	+
II1(brother)	33	55.7↑	15.8↑	31.4↑	0.41	3.75	0.92↓	1.05↓	0.88	1.22↓	+
II3(husband)	31	36.1	10	17.3	0.19	2.88	2.99	2.98	1	3.01	–
III1 (daughter)	7	54.3↑	14.9↑	30.9↑	0.39	4	0.95↓	0.99↓	0.96	1.10↓	+
Reference range		26.6–43.6	7.7–10.5	14.5–19.2	< 0.50	< 5.00	2.00–4.00	2.00–4.00	> 0.7	2.00–4.00	–

APTT: activated partial thromboplastin time; PT: prothrombin time; TT: thrombin time; D-D: D-dimer; FDPs: fibrin(ogen) degradation products; Fg:C: fibrinogen activity; Fg:Ag: fibrinogen antigen.

### Thrombin-induced Fg polymerization assay

**Figure 2:**
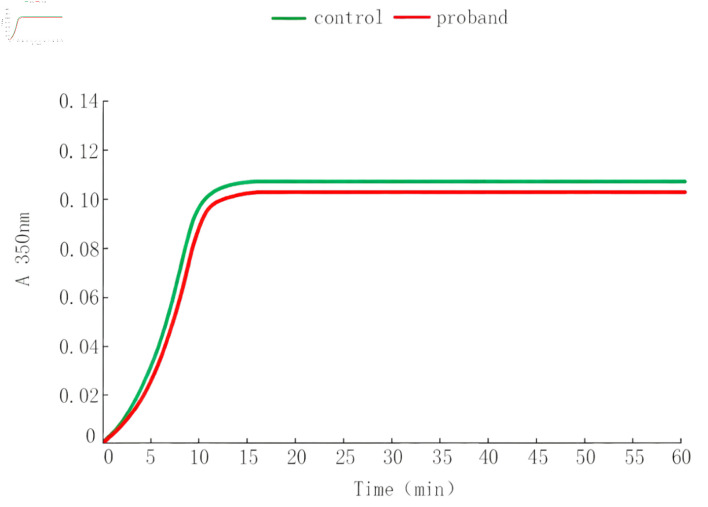
Thrombin-induced fibrinogen polymerization curves.

As shown in Table 3, key parameters including LT, Vmax, and Amax at 350 nm were measured. The proband’s LT was 2.7 ± 0.4 min, which was similar to the control mean value of 2.5 ± 0.3 min. Vmax was 24.8 ± 2.5 mAbs/min in the proband compared with 25.5 ± 2.1 mAbs/min in controls. Amax was 0.109 ± 0.012 Abs in the proband and 0.107 ± 0.010 Abs in controls. All parameters of the proband were within the control range. Consistent with these quantitative findings, the polymerization curves in Figure 2 showed almost complete overlap between the proband and the control, visually confirming that Fg polymerization was not impaired under standardized conditions.

**Table 3:** Results of Thrombin-Induced Fibrinogen Polymerization Assay

Parameter	Control	Proband	Unit
LT	2.5 ± 0.3	2.7 ± 0.4	min
Vmax	25.5 ± 2.1	24.8 ± 2.5	mAbs/min
Amax	0.125 ± 0.010	0.122 ± 0.012	Abs (350 nm)

LT: lag time; Vmax: maximum slope; Amax: maximum absorbance.

### TEG

TEG analysis revealed a distinct hypocoagulable profile in the proband. Key parameters indicated impaired Fg contribution: the K was prolonged to 6.3 min, the α was decreased to 30.0°, and the MA was reduced to 40.5 mm. The overall CI was markedly low at –8.5, consistent with a systemic hypocoagulable state. In contrast, the R, LY30, and EPL were within normal limits, indicating normal initial coagulation factor activity and fibrinolysis. A similar pattern of abnormal TEG parameters—prolonged K time and decreased α angle, MA, and CI—was observed in the proband’s mother, brother, and daughter, while their R, LY30, and EPL values remained normal. All TEG parameters for the proband’s father and husband fell within the normal reference ranges (Table 4).

**Table 4:** Thrombelastography Test Results of the Family With Hereditary Fibrinogen Deficiency

	R (min)	K (min)	α (°)	MA (mm)	CI	LY30 (%)	EPL (%)
II2 (proband)	7.7	6.3↑	30.0↓	40.5↓	–8.5↓	6.0	14.2
I1 (father)	7.4	2.2	62.7	68.2	–0.91	4.5	13.0
I2 (mother)	8.9	5.7↑	31.4↓	41.2↓	–6.3↓	5.3	6.9
II1(brother)	8.1	5.5↑	34.1↓	39.3↓	–6.8↓	3.1	10.9
II3 (husband)	9.3	2.5	60.5	70.0	0.7	5.7	8.5
III1 (daughter)	8.0	5.2↑	31.5↓	44.1↓	–5.2↓	4.1	4.9
Reference range	5.0–10.0	1.0–3.0	50.0–70.0	53.0–72.0	–3.0–3.0	0.0–8.0	0.0–15.0

R: reaction time; K: kinetics time; α: alpha angle; MA: maximum amplitude; CI: coagulation index; LY30: the percentage of clot lysis at 30 min; EPL: estimated percent lysis.

### Genetic sequencing

**Figure 3:**
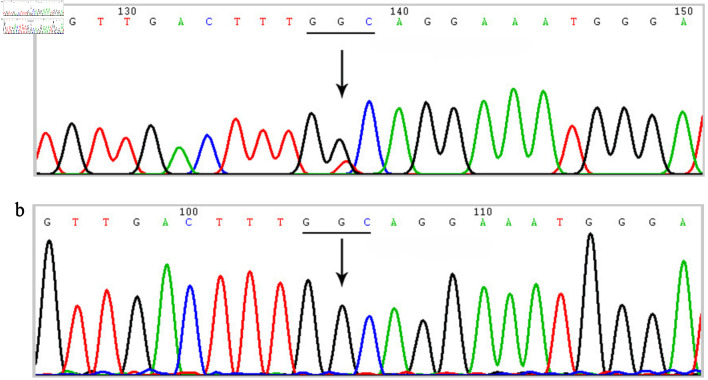
Sanger sequencing chromatograms of the *FGB* gene. (a) Sequencing result of the proband showing a heterozygous missense variant c.878G>T (p.Gly293Val) in exon 6. The arrow indicates the nucleotide substitution (G>T). (b) Representative wild-type sequence at the same locus. Gly: glycine; Val: valine.

Sanger sequencing of the *FGB* gene identified a heterozygous missense variant in the proband: c.878G>T in exon 6, resulting in the amino acid substitution p.Gly293Val (Fig. 3a). Segregation analysis within the family confirmed that the same heterozygous c.878G>T variant was present in her symptomatic mother, brother, and daughter. In contrast, her father and husband were homozygous for the wild-type allele at this locus (Fig. 3b).

### Amino acid conservation analysis

**Figure 4:**
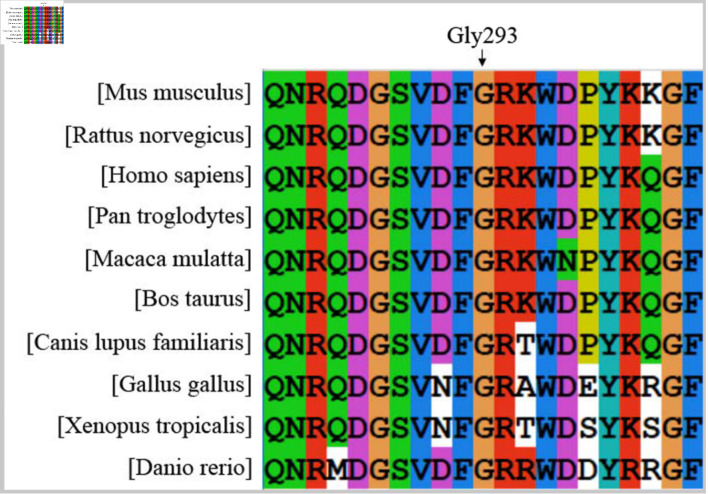
Evolutionary conservation analysis of the Gly293 residue in the fibrinogen Bβ chain. Gly: glycine.

Multiple sequence alignment across 10 vertebrate species revealed that the Gly residue at position 293 of the Fg Bβ chain (Gly293) was completely conserved (Fig. 4), indicating strong evolutionary constraint at this site.

### Bioinformatic analysis of variant

Computational prediction of the functional impact of the p.Gly293Val variant yielded concordant deleterious outcomes across multiple algorithms: MutationTaster (“disease causing”), PolyPhen-2 (“possibly damaging”), PROVEAN (“deleterious”), and I-Mutant 2.0 (“stability decrease”).

### Structural modeling of amino acid variant

**Figure 5:**
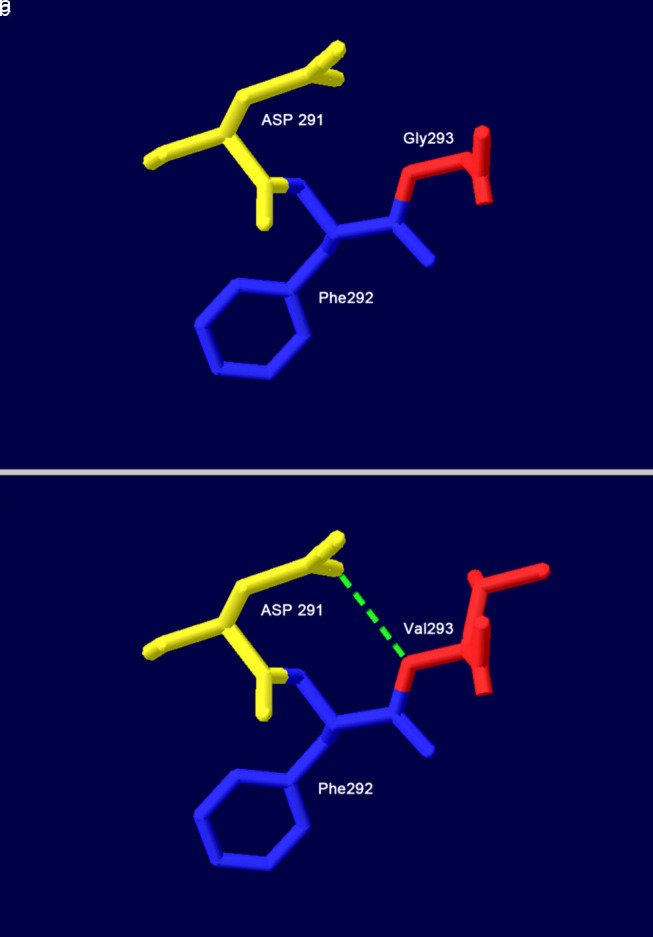
Structural models of the wild-type and mutant fibrinogen Bβ chain around residue 293. (a) Wild-type Gly293. (b) Mutant-type Val293. All structures are displayed in cartoon representation with key residues shown as sticks. Green dashed lines indicate hydrogen bonds. Asp: aspartic acid; Gly: glycine; Phe: phenylalanine; Val: valine.

Molecular modeling of the wild-type Bβ chain confirmed that Gly293, located in a solvent-accessible loop region, does not participate in stabilizing hydrogen bonds with surrounding residues (Fig. 5a). Substitution with valine (Val) introduces a bulky, hydrophobic isopropyl side chain. This alteration leads to a significant local conformational rearrangement. Notably, the Val293 side chain forms a new hydrogen bond with the backbone carbonyl of aspartic acid (Asp) 291 (Fig. 5b). The introduction of this novel interaction, combined with the steric bulk of Val, is predicted to distort the native local backbone geometry and potentially affect the stability or functional dynamics of this region.

### Pathogenicity assessment of the identified variant

Based on the ACMG guidelines, the *FGB* variant c.878G>T (p.Gly293Val) was classified as “likely pathogenic.” This classification was supported by the following evidence: PS3: functional assays, including coagulation studies and TEG, demonstrated a significant functional impairment consistent with hypofibrinogenemia, in all affected carriers. PM1: the variant is located at a highly evolutionarily conserved residue (Gly293) within a structurally sensitive solvent-accessible loop region of the Fg Bβ chain. PM2: the variant is absent from the gnomAD population database. PP1: the variant co-segregated with the abnormal coagulation phenotype in four affected family members across two generations. PP3: multiple computational tools (MutationTaster, PolyPhen-2, PROVEAN, I-Mutant 2.0) consistently predicted a damaging effect on protein function or stability. PP4: the proband and affected family members exhibited a specific laboratory phenotype of isolated prolonged TT with proportional reductions in both Fg:C and Fg:Ag levels, which is characteristic of type I Fg deficiency.

## Discussion

This study identified a novel heterozygous *FGB* variant, c.878G>T (p.Gly293Val), co-segregating with type I hypofibrinogenemia in a Chinese pedigree. The proband and affected family members exhibited the characteristic laboratory triad of prolonged TT, concordantly reduced Fg:C and Fg:Ag levels (with an Fg:C-Clauss/Fg:C-PT-der ratio of 0.79), and a hypocoagulable TEG profile, while thrombin-induced Fg polymerization remained normal. Integrated computational and structural analyses further supported the deleterious impact of the p.Gly293Val substitution. This variant was absent from population databases and classified as likely pathogenic according to the ACMG guidelines. Our findings expand the mutational spectrum of FGB-related hypofibrinogenemia and provide novel insights into genotype-phenotype correlations.

The p.Gly293Val variant introduces a bulky Val side chain into a solvent-accessible loop of the Fg Bβ chain. Structural modeling predicted that this substitution disrupts the local backbone geometry and forms a novel hydrogen bond, potentially altering protein conformation. Interestingly, despite this predicted structural perturbation, the Fg polymerization kinetics in patient plasma were normal after standardizing the concentration. This observation suggests that the primary pathogenic mechanism is not a gross functional defect in Fb clot formation *per se*, but rather a defect that manifests earlier in the Fg lifecycle. This aligns with the type I deficiency phenotype and points towards potential defects in protein folding, intracellular processing, or secretion efficiency—mechanisms commonly implicated in quantitative Fg disorders [9]. Similar mechanisms have been reported for other Bβ-chain variants, such as p.Arg294Gly and p.Arg294Ser, which also affect the same structural region [10]. Our findings suggest that p.Gly293Val may represent a novel structural perturbation mode within this critical calcium-binding and secretion-associated domain. The location of Gly293 outside the canonical functional domains (thrombin cleavage sites, polymerization knobs/holes, FXIII cross-linking sites) may explain the preserved intrinsic function of the secreted, albeit reduced, protein [11].

Gly293 is located in a critical region of the Bβ chain. Structural studies indicate that this residue, along with Asp291, Asp428, and Glu158 on the γ chain, forms part of a conserved calcium-binding site that anchors the Bβ chain D-domain to the coiled-coil, playing a key role in molecular stability and secretion [12]. This region is characterized by five β-strands essential for secretion [13]. Notably, variants affecting the adjacent residue Arg294 have been well documented to cause hypofibrinogenemia through distinct mechanisms. The p.Arg294Gly variant is reported to disrupt this calcium-binding site and the local β-strand structure, impairing secretion [14]. In contrast, the p.Arg294Ser variant is proposed to act primarily by altering a key hydrogen bond with Asp291 [10]. Our modeling suggests that p.Gly293Val, while adjacent to these residues, may exert its effect through steric clash rather than direct disruption of calcium binding or the same hydrogen bond network, representing a novel pathogenic mechanism at this locus. Unlike p.Arg294Gly (which disrupts calcium binding) and p.Arg294Ser (which alters hydrogen bonding), p.Gly293Val appears to primarily perturb local backbone geometry, highlighting the structural sensitivity of this region.

The clinical phenotype in this family was mild and consistent with heterozygous type I deficiency, primarily manifesting as menorrhagia and easy bruising, without severe spontaneous hemorrhage or thrombosis. This correlates well with the residual plasma Fg levels of approximately 1.0 g/L in affected individuals. Our data reinforce the established direct relationship between the level of circulating functional Fg and bleeding risk. The TEG profile, showing a prolonged K time, reduced α angle and MA with a normal R time, perfectly reflects the isolated deficiency of Fg, confirming TEG’s utility in characterizing the functional consequence of such defects.

The c.878G>T (p.Gly293Val) variant has not been previously reported in association with Fg disorders. Its discovery in a Chinese pedigree contributes to the growing catalog of FGB variants and underscores the genetic heterogeneity underlying inherited hypofibrinogenemia. The perfect co-segregation of the variant with the laboratory phenotype across four individuals provides strong genetic evidence for its pathogenicity. This is further bolstered by its absence in control populations and unanimous damaging predictions from multiple *in silico* tools.

A limitation of this study is the lack of direct *in vitro* expression studies (e.g., recombinant protein expression in cell models) to conclusively demonstrate the impact of the variant on Fg biosynthesis and secretion. Future studies employing such approaches would be valuable to confirm the hypothesized defect in protein processing or stability.

In conclusion, we report the first identification and characterization of the *FGB* p.Gly293Val variant as a novel cause of autosomal dominant hypofibrinogenemia. Our comprehensive analysis—spanning detailed phenotyping, functional global hemostasis assays, and *in silico* structural predictions—provides a robust framework for variant interpretation. These findings aid in the molecular diagnosis and genetic counseling of affected families and enhance our understanding of structure-function relationships in the Fg molecule.

## Data Availability

The authors declare that data supporting the findings of this study are available within the article.

## Abbreviations

Fg: fibrinogen
Fg: Ag, Fg antigen
Fg: C, Fg functional activity
FpA: fibrinopeptides A
FpB: fibrinopeptides B
FXIIIa: activated factor XIII
TEG: thromboelastography
PPP: platelet-poor plasma
APTT: activated partial thromboplastin time
PT: prothrombin time
TT: thrombin time
FDPs: Fg/Fb degradation products
LT: lag time
Vmax: maximum slope
Amax: maximum absorbance
R: reaction time
K: kinetics time
α: alpha angle
MA: maximum amplitude
CI: coagulation index
LY30: the percentage of clot lysis at 30 min
EPL: estimated percent lysis
UTRs: untranslated regions
